# Large‐scale early‐wilting response of Central European forests to the 2018 extreme drought

**DOI:** 10.1111/gcb.15360

**Published:** 2020-10-22

**Authors:** Philipp Brun, Achilleas Psomas, Christian Ginzler, Wilfried Thuiller, Massimiliano Zappa, Niklaus E. Zimmermann

**Affiliations:** ^1^ Swiss Federal Research Institute (WSL) Birmensdorf Switzerland; ^2^ Univ. Grenoble Alpes, CNRS, Univ. Savoie Mont Blanc, LECA, Laboratoire d'Écologie Alpine Grenoble France

**Keywords:** climate change, early senescence, European beech, Norway spruce, remote sensing, Sentinel‐2, tree mortality

## Abstract

The combination of drought and heat affects forest ecosystems by deteriorating the health of trees, which can lead to large‐scale die‐offs with consequences on biodiversity, the carbon cycle, and wood production. It is thus crucial to understand how drought events affect tree health and which factors determine forest susceptibility and resilience. We analyze the response of Central European forests to the 2018 summer drought with 10 × 10 m satellite observations. By associating time‐series statistics of the Normalized Difference Vegetation Index (NDVI) with visually classified observations of early wilting, we show that the drought led to early leaf‐shedding across 21,500 ± 2,800 km^2^, in particular in central and eastern Germany and in the Czech Republic. High temperatures and low precipitation, especially in August, mostly explained these large‐scale patterns, with small‐ to medium‐sized trees, steep slopes, and shallow soils being important regional risk factors. Early wilting revealed a lasting impact on forest productivity, with affected trees showing reduced greenness in the following spring. Our approach reliably detects early wilting at the resolution of large individual crowns and links it to key environmental drivers. It provides a sound basis to monitor and forecast early‐wilting responses that may follow the droughts of the coming decades.

## Graphical abstract

We analyze the response of Central European forests to the 2018 summer drought with 10 × 10 m satellite observations. By associating time‐series statistics of the Normalized Difference Vegetation Index with aerial observations of early wilting, we show that the drought led to early leaf‐shedding across 21,500 ± 2,800 km^2^, in particular in Germany and the Czech Republic. Temperature and precipitation mostly explained these large‐scale patterns, with small to medium‐sized trees, steep slopes, and shallow soils being regional risk factors. Our approach provides a sound basis to monitor and forecast early wilting that may follow the droughts of the coming decades.

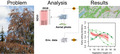

## 1 INTRODUCTION

**FIGURE 1:**
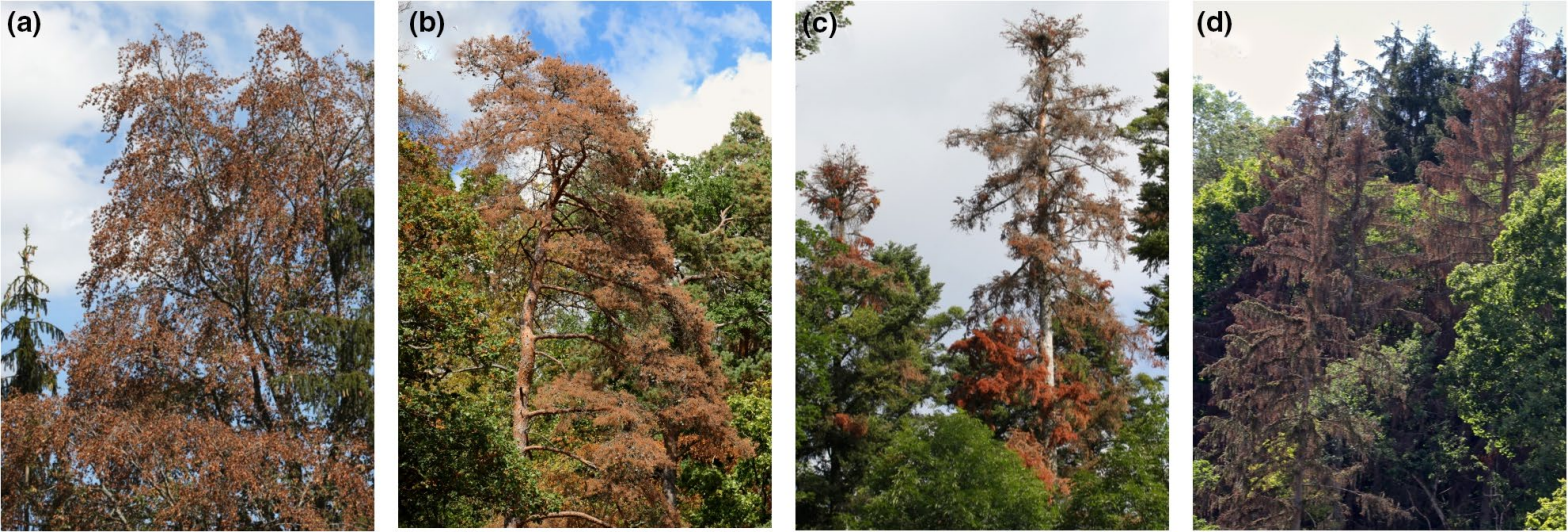
Examples of early‐wilting trees. Early‐wilting responses in the ecologically and economically important species European beech (a), Scots pine (b), silver fir (c), and Norway spruce (d). Photos are courtesy of A. Rigling

During the summer 2018, Central Europe experienced the most extreme drought and heat wave on record, exceeding even the millennial drought of 2003 (Buras et al., 2020; Schuldt et al., 2020). While large, positive temperature anomalies struck the entire continent north of 45°N, Central Europe also suffered from major deficits in the climatic water balance (Buras et al., 2020). Across Germany, Austria, and Switzerland, growing‐season temperatures reached highest values since 1900 (+3.3°C above the 1961–1990 average) while growing‐season precipitation was at its fifth‐lowest level, leading to the highest vapor pressure deficit in at least 118 years (Schuldt et al., 2020; see also Figure S1). These conditions resulted in severe water‐stress symptoms in most economically and ecologically important tree species, including leaf discoloration, premature leaf‐shedding, and mortality (Figure 1; see Schuldt et al., 2020 for more information). And these stress symptoms, in turn, caused significant public attention: in German‐speaking Switzerland c. 900 newspaper articles were written about the topic during 2018 (Figure S2). Nevertheless, beyond ample anecdotal evidence on the occurrence of water‐stress symptoms in trees, little information exists on their spatial distribution and underlying environmental drivers. Here, we developed an approach that exploits state‐of‐the‐art remote‐sensing data to address these issues.

Remote‐sensing data are commonly employed to quantify the spatial patterns of drought impact on forests, but the focus on proxy variables with coarse spatial and/or temporal resolution so far largely prohibited explicit detection of water‐stress symptoms across large areas. Remote‐sensing estimates of drought impact on forests usually focus on productivity, which is approximated by vegetation indices (Buras et al., 2020; Schuldt et al., 2020; Xu et al., 2011) or modeled based on remotely sensed information (Ciais et al., 2005), or they focus on tree mortality, which is approximated by indices like the fraction of non‐photosynthetically active vegetation (e.g., Anderegg et al., 2019). For the Central European 2018 summer drought, two studies have compared the mid‐August Normalized Difference Vegetation Index (NDVI) to previous years at 231 m resolution, indicating that across most of Germany, Austria, and Switzerland the 2018 values were among the lowest since 2000 (Buras et al., 2020; Schuldt et al., 2020). While these assessments provide useful information on productivity, they are less powerful to identify water‐stress symptoms at the individual tree‐crown level which are typically patchy (Figure 1) and often blurred in 250 × 250 m pixels (McDowell et al., 2015). To understand how drought affects tree health, high‐resolution data are needed and water‐stress symptoms should be quantified as directly as possible. To this end, the stress symptoms considered need to be clearly visible from space.

A symptom of severe water stress that can be readily detected from space is “early wilting.” We define early wilting as leaf discoloration and leaf‐shedding occurring before the end of September, which is distinctly before the normal onset of senescence in the dominant deciduous tree species of Central Europe (mid‐October). Early wilting occurs in both deciduous and evergreen trees (Figure 1). In deciduous trees it is often associated with an early entrance into the resting phase during the cold/dry season but it may also be caused by mortality, while in evergreen trees it is always caused by mortality. Early wilting produces a sudden change in canopy color and leaves a clear signal in high‐resolution observations from space.

Investigations of the main environmental drivers of early wilting are challenged by the necessity to compare processes that act on vastly different scales, some remain constant across many kilometers while others vary for each individual tree (Choat et al., 2018; Hartmann et al., 2018). Climate anomalies, which form the very definition of drought, and climate are key drivers of early wilting, with patterns that vary regionally. Precipitation in Central Europe, for example, is highest in and around the European Alps and gradually decreases toward lower elevations in the north, reaching a minimum in eastern Germany (Figure S1). Studying the effect of climatic factors therefore requires comparably large spatial extents to capture sufficient variation, and thus, due to computational constraints, relatively crude grain sizes. However, ultimately water‐stress symptoms result from soil‐water scarcity rather than from precipitation or temperature (Allen et al., 2015). And beyond climate, soil‐water scarcity is influenced by soil conditions, terrain, and competition for water among neighboring plants (Gleason et al., 2017). Similarly, the size and position of individual trees determine their susceptibility to wilt early, that is, their canopy height, their rooting depth (Trugman et al., 2018), and their closeness to the forest edge (Baader, 1952). These factors vary at increasingly smaller scales and require environmental data with fine spatial resolution which limits the spatial extents that can be investigated. A comprehensive assessment of the importance of environmental drivers of early wilting needs to account for these scale differences by focusing on multiple spatial scales.

We comprehensively analyzed (a) the patterns; (b) the drivers; and (c) the consequences of early wilting in Central European forests during the 2018 summer drought. We inferred spatial patterns of early wilting *explicitly*, rather than studying proxies, by training random forest models (Breiman, 2001) with visual classifications of orthophotos as response (>300,000 classified pixels) and averages, extrema, trends, and change points of NDVI time‐series as predictors. The NDVI time‐series used stemmed from the Sentinel‐2 mission and had a very high resolution of 10 m spatially and 3–5 days temporally (Drusch et al., 2012). We considered three spatial scales to investigate environmental drivers of early wilting. We focused on the finest scale possible, the “regional scale,” to capture the role of local drivers, considering three regions in Switzerland with prevalent early wilting (c. 150 km^2^ each, 10 m grain), and we studied the largest scale possible, the “Central European scale,” to obtain robust relationships with climatic drivers, considering c. 800,000 km^2^ of Central Europe (500 m grain). In addition, we investigated environment–early wilting relationships at an intermediate scale, the “Swiss scale,” focusing on Switzerland (c. 41,000 km^2^, 100 m grain) with its rich availability of environmental data. Finally, we studied the effect of early‐wilting responses on following‐spring greenness. To this end, we assessed how the 2019 May/June NDVI changed relative to the previous year in pixels affected by early wilting compared to non‐affected pixels. Based on this set up, we addressed the following research questions.

1. What were extent and spatial patterns of early wilting across Central Europe in 2018?
2. How is early wilting related to climate, soil conditions, terrain, and vegetation structure, and how do these relationships differ across spatial scales?
3. What was the effect of early wilting on canopy greenness in May/June 2019 and which factors best explained it?

## 2 METHODS

### 2.1 Overview

**FIGURE 2:**
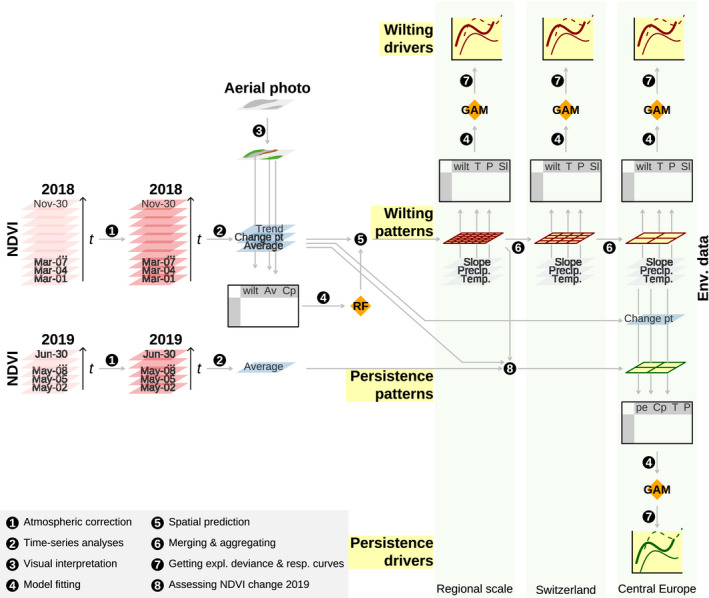
Schematic illustration of the analyses conducted in this study. Black circles indicate data transformations and arrows indicate flow of information. Results are highlighted in yellow. Greenish shades in the background represent the different scales analyzed: regional scale represents three regions of c. 150 km^2^ with 10 m grain; Switzerland covers c. 41,000 km^2^ with 100 m grain; and Central Europe covers c. 800,000 km^2^ with 500 m grain. Av, average; Cp and Change pt, change point; GAM, generalized additive model; P and Precip., precipitation; pe, persistence, that is, the effect of early wilting on following‐spring greenness; RF, random forest; Sl, slope; Tr, trend; T and Temp., temperature; wilt, early wilting

We conducted the following analysis steps to identify the spatial patterns of early wilting, its main environmental drivers, and its effect on following‐spring greenness (Figure 2): to infer spatial patterns of early wilting, we first derived summary statistics of the NDVI time‐series covering March to November 2018 (averages and extrema of different periods, and change points and trends from May to September). Next, we visually determined whether trees have wilted early for 1,022 polygons which covered more than 304,000 Sentinel‐2 pixels and were spread across the study area, using true‐color remote‐sensing images of <1–5 m resolution. Combining visual interpretations and time‐series statistics, we then trained a random forest classifier and used it to predict early‐wilting presence across Central Europe. To identify the key drivers of early wilting we took stratified samples of classified pixels and combined them with data on vegetation structure, terrain, soil, climate, and climate anomalies. Then, we investigated the strength and nature of relationships between early wilting and these drivers at the regional, the Swiss, and the Central European scale. To investigate the effect on following‐spring greenness, we assessed to which extent the NDVI difference between patches affected by early wilting and non‐affected patches changed between May/June 2018 and May/June 2019.

### 2.2 Data

#### 2.2.1 NDVI data

The NDVI data were derived from Level 1 observations of the Sentinel‐2 instruments (Drusch et al., 2012). Sentinel‐2 observations had a 10 m spatial resolution and a 3‐ to 5‐day temporal resolution. We used Sentinel‐2 observations from 87 tiles covering our study area (Figure S3) during the periods March 1 to November 30, 2018 and May 1 to June 30, 2019.

#### 2.2.2 True color imagery for visual identification of early‐wilting presence

We used two data sources to visually determine whether or not early wilting occurred: high‐resolution and very high‐resolution remotely sensed images (the latter referred to as orthophotos in this study). High‐resolution images were obtained from PLANET (www.planet.com). PLANET provides daily images of 3–5 m spatial resolution, allowing the study of temporal changes under non‐cloudy conditions. Orthophotos from Google Earth (https://www.google.com/intl/en/earth/) had sub‐meter spatial resolution, allowing the observation of individual trees. However, Google Earth orthophotos taken within the critical period (end of July to mid‐October 2018) were only available for about a fifth of the study area. Based on these preconditions, we created two sets of classification data: one screening the study area randomly for availability of accurate Google Earth orthophotos, and one based on evenly distributed sampling across the entire study area, considering only larger patches of clearly visible early‐wilted trees when we had to rely on PLANET data (see below).

#### 2.2.3 Weather data

We estimated averages and anomalies of maximum temperature and precipitation at the Swiss and the Central European scale. At the Swiss scale, averages and anomalies of maximum temperature and precipitation were calculated using Daymet (Thornton et al., 1997) to interpolate daily weather conditions from observations of MeteoSwiss weather stations to the study area. We first averaged the original, daily Daymet layers with 100 m resolution to monthly means. Then, we estimated climatological means based on the period 1981–2010 as well as anomalies for 2018. Since no a priori knowledge on the most meaningful period to consider existed, we generated estimates for all combinations of single and consecutive months between April and August, that is, April, May, June, July, August, April/May, May/June, June/July, July/August, April–June, May–July, June–August, April–July, May–August, and April–August. Furthermore, we estimated anomalies for the recent Western and Central European drought period from June 2016 to July 2017 (García‐Herrera et al., 2019). For temperature, we averaged measurements over the periods considered and calculated anomalies in absolute numbers as °C deviation from the climatological mean. For precipitation, we summed measurements over the periods considered and calculated anomalies in relative numbers, as percentage of the precipitation sum. At the Central European scale, data for maximum temperature and precipitation were extracted from the Climatologies at High resolution for the Earth's Land Surface Areas (CHELSA) initiative (Karger et al., 2017) with an original daily resolution and a spatial grain of 30 arc‐sec. CHELSA data are generated by mechanistically downscaling a leading reanalysis product that models the state of the global atmosphere (Karger et al., 2017). For Switzerland, however, CHELSA data have a scarcer data basis (40–45 grid points vs. >400 measurement stations) and are downscaled to a cruder digital elevation model than Daymet interpolations are. Average Pearson correlation coefficients between the two data products are 0.95, 0.90, 0.48, and 0.42 for maximum temperature means, precipitation means, maximum temperature 2018 anomalies, and precipitation 2018 anomalies, respectively. After projecting CHELSA data to an equal area grid for Europe (EPSG 3035) with 1,000 m resolution, we derived the same statistics as we calculated at the Swiss scale.

#### 2.2.4 Soil data

The soil data used consisted of information on soil structural properties for both, Switzerland and Central Europe, as well as information on climatological soil moisture for Switzerland. At the Swiss scale, data on soil structural properties were obtained from the “Bodeneignungskarte” of the Federal Statistical Office available as ESRI shapefile. We considered the indices for “soil depth,” “water permeability,” “water holding capacity,” and “coarse fragment content” as proxies for rooting depth, hydraulic conductivity, storage capacity, and coarse soil content, respectively, rasterized the shapes at 10 m resolution for regional‐scale analyses, and aggregated the resulting layers by average to 100 × 100 m for the Switzerland‐scale analysis. At the scale of Central Europe, we used data on soil structural properties from the European Soil Data Centre. We used information on water content at field capacity (to represent storage capacity) as well as soil hydraulic conductivity from the maps of indicators of soil hydraulic properties for Europe with 250 × 250 m resolution (Tóth et al., 2015). After calculating profile averages from the seven soil layers distinguished, we aggregated the information by average to 500 × 500 m. Finally, we used gravel content (to represent coarse soil content) and rooting depth from the European Soil Database (Hiederer, 2013) with 1,000 × 1,000 m original resolution. Soil moisture averages and anomalies were estimated for Switzerland based on data from the hydrological model PREVAH (Viviroli et al., 2009), as presented in Speich et al. (2015). Daily gridded data on soil water saturation at 500 m resolution were available for the years 2016–2018. We calculated soil moisture baseline values from the years 2016–2017 and derived anomalies for 2018 relatively, as percentages of the baseline values. Soil moisture statistics were calculated for the same monthly periods as considered for maximum temperature and precipitation statistics.

#### 2.2.5 Terrain data

We used terrain data from two different data sources to link early‐wilting patterns with environmental drivers at the regional, the Swiss, and the Central European scale. For the Swiss analysis, we derived four terrain variables from the SwissAlti3D digital elevation model that is maintained by the Swiss Federal Office of Topography (swisstopo). We aggregated this elevation model from the original 2 meter resolution by averaging to 10 × 10 m to match it with the resolution of the Sentinel‐2 observations. In addition to using the aggregated elevation layer directly, we derived from it slope, north/south component of exposition, and the terrain wetness index (TWI), that is, the likelihood of the soil to receive lateral water flow from the surrounding upslope area (Beven & Kirkby, 1979). For the Swiss‐scale analysis, we further aggregated elevation, slope, and TWI by average to 100 m resolution and deduced the north/south component of exposition from the aggregated elevation raster. For Central Europe, we used the EU‐DEM digital elevation model from the European Environment Agency with 25 m resolution. From this data set, we deduced slope estimates and aggregated both, elevation and slope, to 500 m resolution by averaging. From the aggregated elevation raster, we again deduced the north/south component of exposition. Finally, at the Central European scale we used TWI estimates from Marthews et al. (2015) with 15 arc‐sec original spatial resolution, which we reprojected to ESPG 3035 with 500 m resolution.

#### 2.2.6 Vegetation data

We considered forest masks and forest mixture at all scales, and vegetation height mean, vegetation height standard deviation, and distance to forest edge for Switzerland only. A 1 × 1 m forest mask for Switzerland (Waser et al., 2015) was used to derive regional‐scale and Swiss‐scale information. We aggregated this mask to 10 m resolution by majority vote. At the Central European scale, we derived a forest mask based on the Tree Cover Density map 2015 from Copernicus (https://land.copernicus.eu/), which has an original resolution of 20 m. Areas with less than 80% tree coverage were considered non‐forested, and removed from the analyses. For forest mixture (fraction of broadleaved trees relative to needle‐leaved trees), we used the tree‐type map for Switzerland (Waser et al., 2017) with 25 m original resolution at the regional and the Swiss scale. At the Central European scale, we considered the Forest Type map 2015 from Copernicus with 20 m original resolution. Mean and standard deviation of vegetation height were estimated from a 1 × 1 m vegetation canopy height model for Switzerland (Ginzler & Hobi, 2015) that we aggregated to 10 × 10 m by average and standard deviation, respectively. Distance to forest edge was calculated from the 10 × 10 m aggregate of the Swiss forest mask. Unless otherwise noted, all layers were aggregated by average to 10, 100, and 500 m, for regional‐scale, Swiss‐scale, and Central European‐scale analyses, respectively. An overview over all data used in this study is given in Table 1.

**TABLE 1:** Overview of the data used in this study

Analysis	Group	Variable names	Deduced variables	Spatial extent	Spatial resolution	Temporal extent	Temporal resolution	Origin^a^
Predicting early‐wilting patterns	Vegetation indices	NDVI	Averages, extrema, change points, trends	Central Europe	10 m	Mar–November 2018 and May/June 2019	3–5 days	Sentinel‐2
	True color images	Orthophotos	Classified occurrence of early wilting	Central Europe (patchy)	<1 m	End of July to mid‐October 2018	—	Google Earth
		High‐resolution images		Central Europe	3–5 m		1 day	PLANET
Studying early‐wilting drivers	Weather	Maximum temperature, precipitation	Averages, anomalies 2018, anomalies 2016/2017	Switzerland	100 m	1981–2010 and 2016–2018	1 day	MeteoSwiss with Daymet interpolation
				Central Europe	1,000 m			CHELSA
	Soil	Rooting depth, hydraulic conductivity, storage capacity, coarse soil content	Averages	Switzerland	10 m^b^	—	—	Federal Statistical Office
				Central Europe	250–1,000 m	—	—	European Soil Data Centre
		Soil moisture	Averages, anomalies 2018	Switzerland	500 m	2016–2018	1 day	Speich et al. (2015)
	Terrain	Elevation	Averages, slope, S/N exposition, TWI	Switzerland	2 m	—	—	swisstopo
				Central Europe	25 m	—	—	European Environment Agency
	Vegetation	Vegetation height	Averages, standard deviations	Switzerland	1 m	—	—	Ginzler and Hobi (2015)
		Forest mixture	Averages	Switzerland	25 m	—	—	Waser et al. (2017)
			Averages	Central Europe	20 m	—	—	Copernicus
		Forest mask	Dist. to forest edge	Switzerland	1 m	—	—	Waser et al. (2015)
				Central Europe	20 m	—	—	Copernicus

^a^ For more information, see main text.

^b^ Original information available as shapefile.

### 2.3 Analyses

#### 2.3.1 Time‐series analyses of NDVI data

We derived averages, extrema, change points, and trends from NDVI time‐series and used them as predictors to infer for each pixel whether or not early wilting was present. To this end, we used the FORCE v2.0 software (Frantz, 2017; https://www.uni‐trier.de/index.php?id=63673). This software allows automated downloading and processing of Sentinel‐2 data, including an improved detection of clouds and cloud shadows (Frantz et al., 2018) as well as conducting various types of time‐series analyses. We ran FORCE with the following settings: we downloaded all tile‐based Level 1 observations within the study area and period if they had a cloud coverage ≤50%. We generated radiometrically consistent Level 2 products by running topographic corrections, atmospheric corrections, and cloud detection, accounting for terrain effects through the EU‐DEM digital elevation model. For the Central European analysis, the data were projected to EPSG 3035; for the Swiss analysis, they were projected to the CH1903+/LV95 coordinate reference system (EPSG 2056). Then, we estimated NDVI and ran time‐series analyses for five periods. For May 1 to September 30, 2018, we ran a “Change, Aftereffect, Trend”‐analysis (Hird et al., 2016). This analysis identifies timing and magnitude of the most pronounced change point in the time‐series and estimates linear trends before and after this change point, as well as for the entire time‐series. To better discriminate wilted grassland patches that were not masked off by the forest masks, we also analyzed two short time‐series at the beginning (March 1–April 30) and at the end (October 1–November 30) of the growing season 2018, only estimating NDVI extrema, mean, and standard deviation. Similarly, we estimated NDVI extrema, mean, and standard deviation for the periods May 1 to June 30, 2018 and 2019 to investigate the effect of early wilting on following‐spring greenness. Defining the beginning of the growing season between March 1 and April 30 led us to exclude pixels with persistent snow coverage during this period from our analysis, resulting in the partial removal of high‐altitude forests. Due to this exclusion, our analysis did not cover 100% of forests across the study area.

#### 2.3.2 Visual identification of early wilting

We created two sets of training data and one set of test data by visually interpreting PLANET and Google Earth images. For the first set of training data, we sampled polygons of early‐wilting presence and absence in the proximity of c. 150 mostly randomly chosen points with coverage of suitable Google Earth orthophotos. In this set, unambiguous identification was the most important criterion and sampling effort could vary from point to point. For the second set, we sampled each of the 87 tiles analyzed (Figure S3), using preliminary classifications to identify locations of likely occurrence of early wilting. Based on PLANET and, where available, Google Earth orthophotos we screened the proximity of such locations for one to three polygons of both early‐wilting presence and absence, whenever the quality of the images allowed for a confident interpretation. Preliminary analyses indicated that classifications are susceptible to false positives from patches with wilted grass, bare soil, or logging activities. We therefore also generated a set of polygons covering such artifacts in a low‐ and a high‐altitude region of Switzerland. All polygons were drawn with the Google Earth Pro software.

Following best practice recommendations (Olofsson et al., 2014), we additionally created a set of high‐quality, independent test data. We randomly picked 3,000 pixels for which our classifier predicted presence of early wilting, and 3,000 pixels for which it predicted absence. For each of these pixels, we assessed whether or not Google Earth imagery existed during the period August 1 to September 30 to infer presence of early wilting or during the period August 25 to October 15 to infer absence. Pixels for which this information existed in a quality that allowed a confident assessment were evaluated independently by three people, using majority votes to decide in ambiguous cases. The resulting data sets are summarized in Figure S4 and Table S1.

#### 2.3.3 Predicting presence/absence of early wilting

We identified the relationship between visually observed early wilting and NDVI time‐series statistics by fitting a random‐forest classifier. Random forest is a powerful machine learning technique based on classification trees (Breiman, 2001; Cutler et al., 2007). As the dependent variable we used the visually labeled pixels of early‐wilting presence (value = 1) and absence or artifacts (value = 0). As explanatory variables, we selected 10 statistics summarizing NDVI time‐series based on relevance, data coverage, and absolute Pearson correlation coefficients no larger than 0.75 (see Results S1 for a detailed justification). The selected set included the NDVI spring minimum, the fall minimum, the spring maximum, the fall maximum, the summer mean, the magnitude of change at the summer change point, the timing of the summer change point, the temporal summer trend, the significance of the temporal summer trend, and the mean absolute error during summer. We extracted these variables for all pixels within the classified polygons and fitted a random forest classifier with 500 trees and a sample size of 75,000 pixels of early‐wilting presence and 20,000 pixels of early‐wilting absence for each tree. Then, we predicted early‐wilting presence/absence across the whole forested area that was fully covered by NDVI time‐series statistics, for both Switzerland and Central Europe. For further analyses at the Swiss and the Central European scale, we aggregated the predictions to 100 and 500 m resolution, respectively, reporting fractions of forested area affected by early wilting. Random forest models were fitted in the R environment (R Development Core Team, 2008), using the package randomForest (Liaw & Wiener, 2002).

#### 2.3.4 Validating early‐wilting predictions

We used our independent test data set to assess the skill of early‐wilting predictions and to estimate the area with early‐wilting presence. Following best practice recommendations (Olofsson et al., 2014), we calculated mean and standard errors of overall accuracy, positive predictive value, negative predictive value, and early‐wilted area, using post‐stratified estimators. We assumed our test data set to be an equal‐probability design (and thus suitable Google Earth imagery to be roughly randomly distributed across the study area) and weighted the error matrix derived from the independent validation by the proportion of area predicted to show early‐wilting presence and absence. Finally, we used the class‐size estimates from the visual interpretation to calculate the proportion of area with early‐wilting presence.

#### 2.3.5 Investigating environment–early wilting relationships

We investigated the strength and the shape of the relationships between early wilting and environmental drivers at the regional, the Swiss, and the Central European scale using generalized additive models (GAMs; Hastie & Tibshirani, 1990). The calibration strength was estimated as explained deviance of univariate and multivariate models fitted with balanced class sizes. At the regional scale, balanced class sizes were obtained by randomly sampling 5,000 forest pixels where early wilting was predicted to be present and 5,000 locations where early wilting was predicted to be absent. At coarser scales, balanced samples were obtained by randomly sampling 10,000 raster cells with complete data coverage and multiplying the number of 10 × 10 m pixels with presence of early wilting in each cell with a constant factor so that across all cells the number of presences was equal to the number of absences. As explanatory variables in the multivariate set we used mean vegetation height, distance to forest edge, forest mixture, slope, south/north exposition, rooting depth, hydraulic conductivity, August soil moisture, August 2018 soil moisture anomaly, maximum temperature of August, August 2018 maximum temperature anomaly, July 2016–June 2017 maximum temperature anomaly, April–August precipitation, August 2018 precipitation anomaly, and July 2016–June 2017 precipitation anomaly. These variables were selected based on ecological relevance, explained deviance as inferred by a preliminary analysis, and limited multicollinearity within the predictor set (see Results S1 for a detailed justification). We ran GAMs assuming binomial error distributions. We kept the model formulas fixed but let the algorithm optimize the degrees of freedom assigned to each smoother. Since soil structural variables for Switzerland were resolved as crude classes, we limited the maximum degrees of freedom of smoothers of all soil structural variables to three (*k* = 3). Response curves were deduced from univariate fits. We fitted each model 100 times based on resampled data and report medians and 95% confidence intervals. We used the R package mgcv (Wood, 2006) to fit GAMs.

#### 2.3.6 Estimating following‐spring effects on greenness

We assessed the effect of early‐wilting responses on following‐spring greenness by comparing the NDVI of forest patches with early‐wilting presence against the NDVI of patches with early‐wilting absence. Within each 500 × 500 m cell, we calculated the median NDVI of pixels with early‐wilting presence and pixels with early‐wilting absence for the periods May/June 2018 and May/June 2019. Then, we calculated NDVI differences between pixels affected and unaffected by early wilting and estimated how these differences changed between 2018 and 2019, which is before and after the drought. Furthermore, we investigated the strength and the shape of the relationships between these following‐spring effects and both environment and severity of early‐wilting responses: analogous to the environment‐early wilting relationships at the Central European scale, we fitted univariate and multivariate GAMs and deduced explained deviance and univariate response curves. In addition to the environmental predictors used to fit environment‐early wilting relationships, we considered time and magnitude of change from the “Change, Aftereffect, Trend” time‐series analysis of May–September 2018 as predictors (see Section 2.3.1 for a description).

## 3 RESULTS

### 3.1 Early‐wilting patterns

**FIGURE 3:**
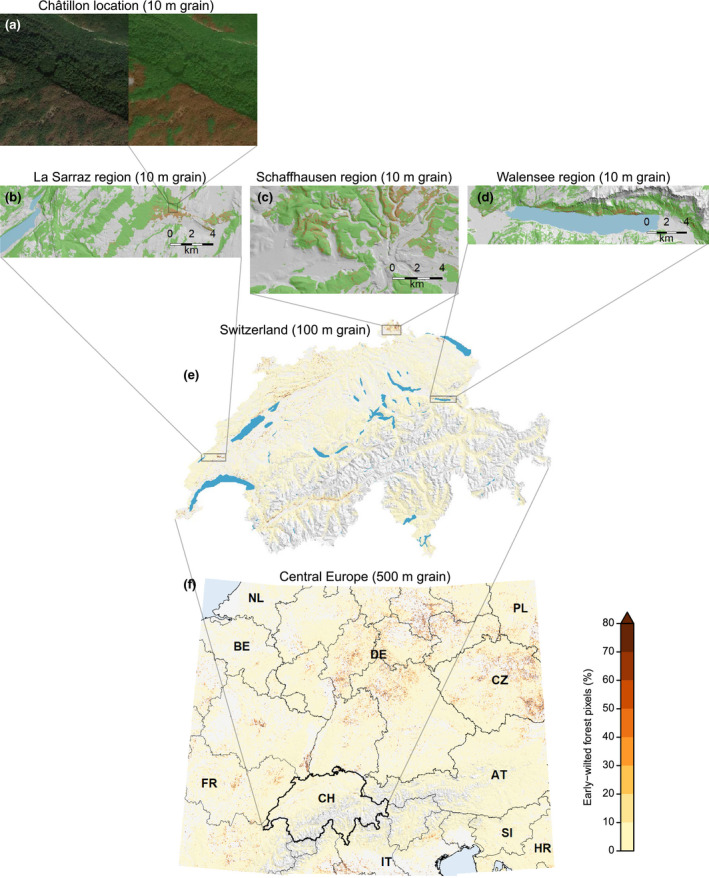
Spatial patterns of early wilting in response to the Central European 2018 summer drought. (a) Google Earth orthophoto on September 25 and model prediction of early‐wilting presence (brown) and absence (green) at Châtillon location. (b)–(d) Early‐wilting presence/absence in the regions “La Sarraz,” “Schaffhausen,” and “Walensee,” respectively. (e) Fractions of early‐wilted forest for Switzerland. (f) Fractions of early‐wilted forest in Central Europe

Early wilting was present in about 11% of the Central European forests investigated. Our random forest classifier achieved an overall accuracy of 0.90 ± 0.014; the positive predictive value was 0.63 ± 0.030; and the negative predictive value was 0.91 ± 0.014. The estimated area affected by early wilting totaled 21,524 ± 2,846 km^2^ or 10.8% of the forested area. Highest prevalence of early wilting was found in central and eastern Germany and in the Czech Republic while lowest prevalence occurred in and around the European Alps. However, early wilting generally befell the entire study area (Figure 3f). Among the most affected political units were the German states of Saxony Anhalt and Thuringia as well as the Czech Republic (Figure S5). Wilted patches were often found on hilltops and crests, and on steep, south‐facing slopes. Yet, larger patches also wilted on flat ground (Figure 3a–d).

### 3.2 Early‐wilting drivers

**FIGURE 4:**
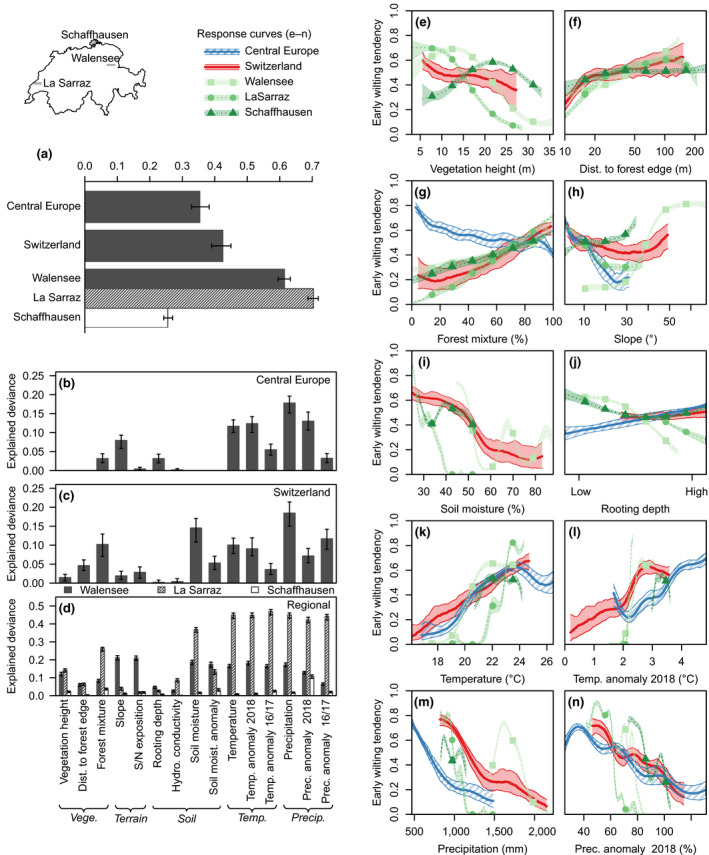
Relationships between environmental drivers and early wilting. Panel (a) shows explained deviance of multivariate models considering full predictor sets (predictors with bars in panels b–d). (b)–(d), Deviance explained by univariate models for Central Europe (b), Switzerland (c), and focal regions (d), respectively. Bars indicate median values of 100 models fitted on resampled data; error bars indicate the 95% confidence intervals. (e)–(n), Univariate response curves representing different regions/scales. Central lines represent median estimates of 100 generalized additive models fitted on resampled data; polygons represent 95% confidence intervals. For distinction of regions/scales see legend on top center. Response curves of further predictors are shown in Figure S7

With 11 predictors available (Figure 4b), at the Central European scale (500 m grain size) we were able to explain 35% of the deviance of early‐wilting patterns (Figure 4a), while at the scale of Switzerland (100 m grain size) the 15 predictors available (Figure 4c) accounted for 42% of the deviance. The explained deviance of the regional models (15 predictors, 10 m grain size) was quite variable. The “Walensee” and “La Sarraz” regions covered rather wide ranges of environmental conditions, and areas affected by early wilting were comparably well confined (Figure 3b–d). In these regions, the explained deviance was well over 50%. The “Schaffhausen” region, on the other hand, covered comparably narrow environmental gradients and contained the most widespread early wilting of Switzerland. In this region, explained deviance was only 26%.

Climate and climate anomalies were major drivers of early wilting overall, and in particular at the Central European scale. At both, the Central European and the Swiss scale, mean precipitation was the most important driver (Figure 4b,c), showing a negative association with early wilting (Figure 4m). Somewhat less critical, maximum temperature was the fourth and fifth most important driver of early wilting at the Central European and Swiss scale, respectively, showing a generally positive relationship with early wilting, except for the warm end of the range across Central Europe (Figure 4k) which was associated with weaker climate anomalies (Figure S1). The 2018 anomalies of precipitation and temperature were particularly critical drivers of early wilting at the Central European scale, where they ranked second and third most important (Figure 4c) and showed negative and positive associations with early wilting, respectively (Figure 4l,n).

The most relevant period for climatic conditions was linked to the timing of the onset of early‐wilting responses and also the severity of the 2016/2017 drought was linked to early‐wilting patterns. Overall, climatic conditions of August best matched the identified wilting patterns (Results S1). However, the starting time of early wilting was not consistent throughout the study area: coinciding with the precipitation anomaly patterns, early wilting tended to start earlier in the northeast of Switzerland than in the northwest (Figure S6). Furthermore, we detected negative and positive responses, respectively, to precipitation and temperature anomalies of the European 2016–2017 drought period at the scale of Switzerland (Figure S7).

Forest mixture and distance to forest edge showed the strongest associations with early wilting at the scale of Switzerland. Early‐wilting tendency increased consistently with fractions of deciduous trees at the Swiss and the regional scale, with tree mixture being the fourth most important predictor at the Swiss scale. Interestingly, at the Central European scale the relationship inverted, and increasing fractions of needle‐leaved trees were associated with an increased early‐wilting tendency (Figure 4g). Also the relative importance of distance to forest edge was higher at the Swiss scale than at regional scales, with early‐wilting tendency increasing with distance to forest edge (Figure 4f).

The responses of early wilting to vegetation height, soil structure, and terrain were strongest at the regional scale, where they often showed a different shape than at larger scales. At the Swiss scale, early‐wilting tendency weakly decreased with increasing vegetation height, while at the regional scale relationships were unimodal, with the highest likelihood of wilting for canopy heights between 10 and 25 m (Figure 4e). At the Central European and Swiss scale early‐wilting tendency mostly declined with slope, reaching a minimum and a subsequent slight increase at the high end of the range. In the “Walensee” region, on the other hand, early‐wilting tendency steeply increased with slope, in particular for steep slopes between 30° and 50° that were barely resolved at larger scales (Figure 4g). As expected, early‐wilting tendency decreased with rooting depth at the regional scales, but surprisingly, at larger scales the relationship inverted to weakly increasing (Figure 4h).

### 3.3 Following‐spring effect on greenness

**FIGURE 5:**
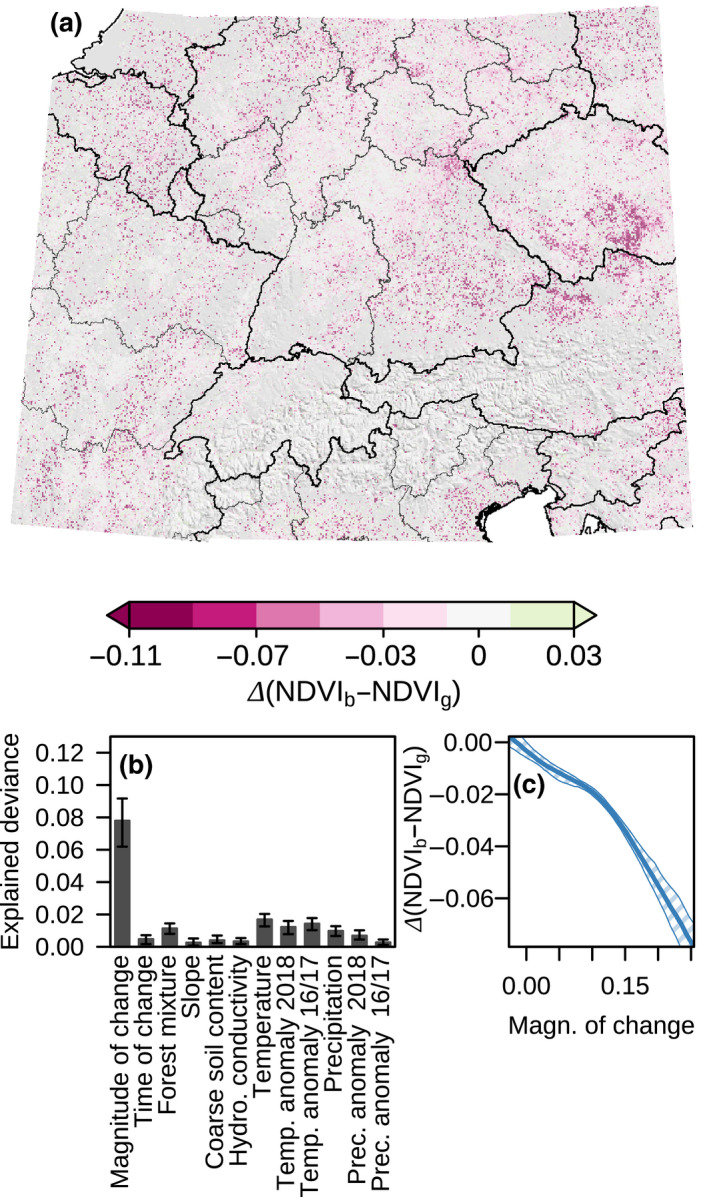
Spatial patterns of following‐spring effect on greenness in early‐wilted forest. (a) Illustration of the 2018–2019 change in May/June Normalized Difference Vegetation Index (NDVI) differences between patches affected by early wilting (NDVI_b_) and non‐affected patches (NDVI_g_) at 500 m grain for Central Europe. (b) Explained deviance of univariate models of the 2018–2019 change in May/June NDVI differences. (c) Response curve of the 2018–2019 change in NDVI difference to the magnitude of NDVI change at the change point of the 2018 NDVI summer time‐series. Central line represents median estimates of 100 generalized additive models fitted on resampled data; polygon represents 95% confidence intervals

Throughout the study area, forests affected by early wilting showed reduced greenness during the spring of 2019 compared to the spring of 2018. On average, the May/June NDVI difference between forest patches affected by early wilting and non‐affected patches increased by 0.015 from 2018 to 2019. The relative NDVI reduction of forest patches affected by early wilting was particularly pronounced in the southern Czech Republic and in northeastern Austria, but declined greenness was also common in eastern France and Belgium (Figure 5a). However, this following‐spring effect on greenness was comparably weakly explained by environment and the severity of early‐wilting responses: the 11 environmental predictors available at the Central European scale (Figure 4b) combined with timing and magnitude of the change point in the 2018 May–September NDVI time‐series only explained 13% of deviance, with magnitude of change being the only predictor explaining >2% alone (Figure 5b,c).

## 4 DISCUSSION

We accurately predicted early‐wilting responses to the 2018 summer drought across Central Europe (overall accuracy = 0.90 ± 0.014) and identified strong links to environmental conditions at three spatial scales, as well as a lasting impact on following‐spring greenness. Overall, early wilting was estimated to be present in about 11% of investigated forests, showing a wide distribution with a main concentration in central and eastern Germany and in the Czech Republic. While drought‐induced tree mortality events in Europe have been observed repeatedly during the past decades (Allen et al., 2010), the impact of the Central European 2018 drought was extraordinarily severe. The wide distribution of immediate early‐wilting symptoms we identified in combination with the premature harvest of 160 million m^3^ of dead wood reported for Germany (BMEL, 2020) suggests that the impact of the Central European 2018 drought may have been more severe than even the most consequential drought listed for Europe between 1960 and 2010 (Allen et al., 2010). This record is held by the northwestern Russian 2004–2006 drought which affected c. 19,000 km^2^ of forest and caused the loss of 208 million m^3^ of timber (Chuprov, 2007; Krotov, 2007; Tsvetkov & Tsvetkov, 2007).

Climatic conditions including soil moisture were overall the most important drivers of early wilting, dominating in particular at the Central European scale. Early‐wilting tendency decreased with means and 2018 anomalies of precipitation and increased with the corresponding statistics for maximum temperature. Among the predictors tested, low precipitation and high temperatures may lead to water stress most directly by reducing soil moisture and increasing vapor pressure deficits (Allen et al., 2015; Seneviratne et al., 2010). Furthermore, leaf temperatures of ≥40°C, as have been measured in Switzerland and Germany during the 2018 drought, are associated with steeply increased water loss through the leaf surface and strongly declined photosynthetic activity (Schuldt et al., 2020). As we expected, at the regional scale the range of climatic conditions was not sufficient for the GAMs to robustly capture the relationships found on the Swiss and the Central European scale, suggesting that it is critical to assess the degree by which climate gradients are covered when quantifying drought impacts in study areas of small spatial extents.

Vegetation height, soil structural properties, and terrain variables were most important at the regional scale. In part, the low importance of these predictors at larger scales may arise from insufficient resolution. To vegetation height, for example, early wilting showed clear unimodal responses at the regional scale, indicating that smallest trees may benefit from lower water loss through transpiration and more efficient water transport through the stem, while largest trees may benefit from deeper rooting systems—at least on the short term (Trugman et al., 2018). At the Swiss scale, the less sharp tree height averages across many crowns may be partly responsible for the weaker, negative relationship identified. Additionally, however, the relevance of vegetation height and soil and terrain properties may be conditional on climate and decrease in study areas of large extents with diverse climates represented. In the “Walensee” region, low rooting depth was associated with increased early‐wilting tendency, presumably because low rooting depth increased the water stress that was already induced by climatic conditions. However, across Switzerland shallow soils often occur at higher elevations with cool and humid climates, which were less affected by the drought (Figure 4). The predictive power of rooting depth alone may therefore be fairly limited at the scale of Switzerland.

Forest mixture was one out of few predictors that were relatively most important at the intermediate Swiss scale, presumably due to indirect associations with climate. At regional and the Swiss scale early‐wilting tendency increased with increasing fractions of broadleaved trees, indicating that European beech—the dominant deciduous tree species—was most susceptible to early wilting in Switzerland. The comparably strong response of early wilting to forest mixture at the Swiss scale may arise because broadleaved trees are more common in warmer regions that were affected more by the drought, whereas Norway spruce, the dominant needle‐leaved species, is native in cooler, higher‐elevation regions (Klimo et al., 2000). The forest mixture‐early wilting relationship inverted at the Central European scale, where needle‐leaved trees appear to have responded with more early wilting to the drought. This inversion may arise because the climatic separation of broadleaved and needle‐leaved trees across Central Europe is much less pronounced. Norway spruce has been cultivated extensively across Central Europe since the 1850s, often also in regions far beyond their original distribution range (Klimo et al., 2000). Many Central European stands of Norway spruce therefore inhabit the warm and dry edge of their ecological niche (Ellenberg, 1988) where they are particularly susceptible to drought.

Early wilting following the 2018 summer drought had lasting consequences during the following growing season. We identified widespread lower NDVI in trees affected by early wilting in May/June 2019 relative to May/June 2018 (Figure 5a), a pattern corresponding to in situ observations of thinner foliation of such trees during spring 2019 (Schuldt et al., 2020). Lasting negative implications on tree health for up to several decades have also been reported in combination with other drought events around the world (Anderegg et al., 2019; Cavin et al., 2013; Trugman et al., 2018) and may be caused by irreparable damages in the water‐conducting tissue of the stems (Trugman et al., 2018).

The high resolution of our data, the numerous statistics derived, and the various environmental predictors considered allowed us to comprehensively study the patterns and drivers of early wilting following the Central European 2018 summer drought, but they also required making several limiting assumptions. First, our data are not free of errors in geo‐positioning. Such errors are most critical when working with high spatial resolutions where they can lead to spatial mismatch. Such mismatches may have lowered the skill of our independent model validation and likely caused errors in masking‐off non‐forested area. We could largely remove false positives from left‐in grassland by including grassland as artifacts in the training data and by adding time‐series statistics for March/April and October/November. Nevertheless, some misclassifications may have remained. Second, we could not make predictions for high‐altitude forests with our approach due to persistent snow coverage in March/April. We therefore ignored them when calculating the proportion of the area affected by early wilting and considered them free of early wilting for mapping. While no forests with snow coverage until April existed in many political units, in Switzerland, where they were most widespread, they constituted 20% of all forests (Figure S5). Third, our set of early‐wilting predictors included many but likely not all relevant drivers. In particular, we lacked information on tree species identity. Central European tree species largely differ in their drought tolerance (Maes et al., 2019; Scherrer et al., 2011; Vanoni et al., 2016) and distinguishing broadleaved and needle‐leaved trees could only mirror these differences to some degree. Finally, the accuracy of our following‐spring NDVI departure estimates may be limited: the weak relationships with 13 diverse predictors tested are likely a consequence of the difficulty to capture the true signal. On the one hand, this may be because predicted early‐wilting probabilities are continuous and splitting pixels within homogeneous 500 × 500 m cells into two classes may overemphasize differences. On the other hand, various processes interfere with the long‐term changes in productivity following early‐wilting responses. Tree species may, for example, recover from drought over different time scales (Trugman et al., 2018); pests may infest weakened trees locally to different extents (Kurz et al., 2008; McDowell et al., 2008); and affected trees may be rapidly harvested, often exposing flushing understory vegetation (McDowell et al., 2015). Such processes may be better controlled for by including more quantitative remote‐sensing information (e.g., light use efficiency or unmixing fractions) or indices that potentially control better for background signals (e.g., fraction of non‐photosynthetically active vegetation or Tasseled Cap Transformation components).

Nevertheless, the early‐wilting patterns identified and their relationships with environmental drivers are robust and provide significant novel insight. The patterns we identified were not related to indirect assessments using coarser sensors: a comparison to mid‐August NDVI quantiles at 1,000 m resolution showed no association with early‐wilting responses (Spearman correlation coefficient of −0.04; Figure S8), emphasizing the necessity to directly target water‐stress symptoms with algorithm‐based detection and high‐resolution data. Similarly, we could explain early‐wilting patterns with environmental conditions in unprecedented detail, and we identified a strong scale dependence of early wilting‐environment relationships. In the coming decades, Central European droughts may occur with increasing frequency (Seneviratne et al., 2012; Zscheischler & Seneviratne, 2017). Our classification algorithm can be used to monitor early‐wilting responses following such droughts, and the environment–early wilting relationships identified here may inform the development of more comprehensive, hierarchical, cross‐scale frameworks to accurately predict water‐stress symptoms before they actually occur.

## AUTHOR CONTRIBUTION

P.B., N.E.Z., A.P., and C.G. conceived the general idea, and designed the study with the help of W.T. M.Z. prepared the soil moisture data and the media analysis. P.B. performed the main analysis and led the writing of the manuscript. All authors significantly interpreted results and contributed to writing and editing.

## Supporting information

Supplementary Material

## Data Availability

The data that support the findings of this study are openly available on Dryad at https://datadryad.org/stash/dataset/doi:10.5061/dryad.d51c5b019.
