# Supplementary material for: Large‐scale early‐wilting response of Central European forests to the 2018 extreme drought
Source: Glob Chang Biol. 2020 Oct 22;26(12):7021–35. doi: 10.1111/gcb.15360 (PMC7756440; doi:10.1111/gcb.15360)
Supplement: Supplementary file 1 — Supplementary Material [file GCB-26-7021-s001.pdf]

Supplementary information for

Large-scale early-wilting response of Central European forests to the 2018 extreme drought

by

Philipp Brun, Achilleas Psomas, Christian Ginzler, Wilfried Thuiller, Massimiliano Zappa, and Niklaus E. Zimmermann

Supplementary Tables & Figures

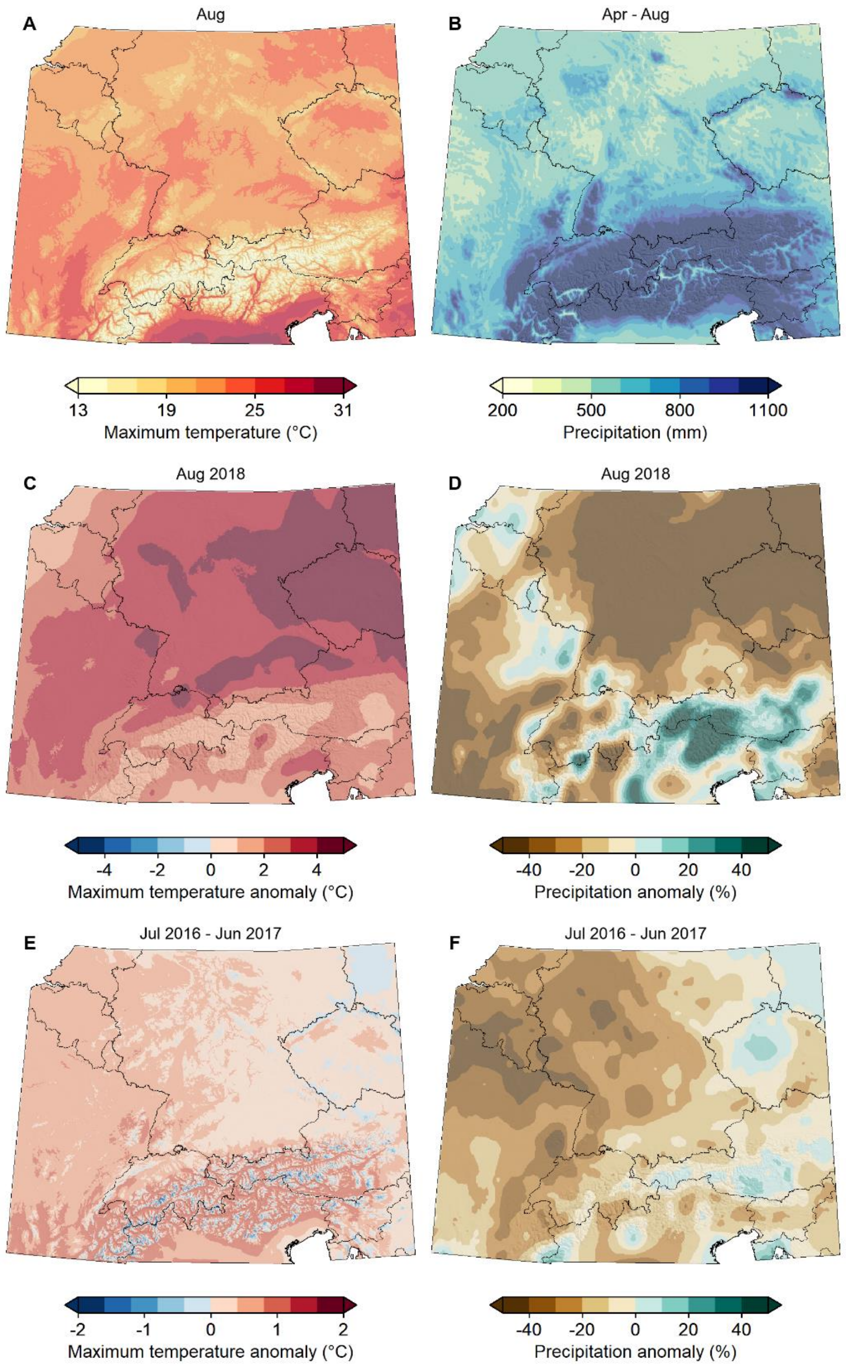

Fig. S1 | Distribution of precipitation and temperature means and anomalies across Central Europe. Panel *A* shows maximum August temperatures. Panel *B* shows average precipitation form April to August. Panels *C* and *D* show maximum temperature and precipitation anomalies, respectively, for August 2018. Panels *E* and *F* show maximum temperature and precipitation anomalies for the July 2016 to June 2017 drought (García-Herrera et al., 2019). Baseline period for climatological means was 1981-2010.

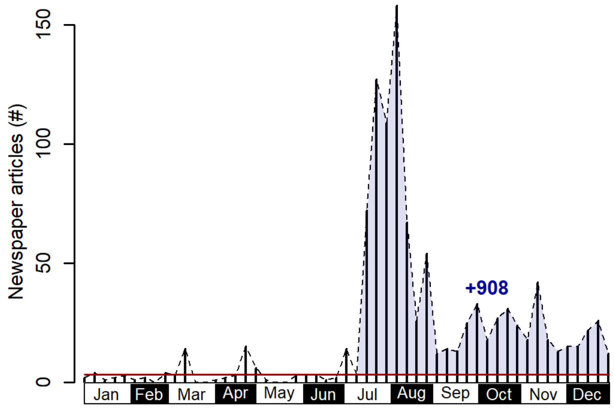

Fig. S2 | Number of newspaper articles related to drought, trees/forests and water stress/wilting responses in the German-speaking part of Switzerland in 2018 (see Supplementary Methods). The red line represents the pre-drought average and blue area represents the surplus of articles in response to the drought.

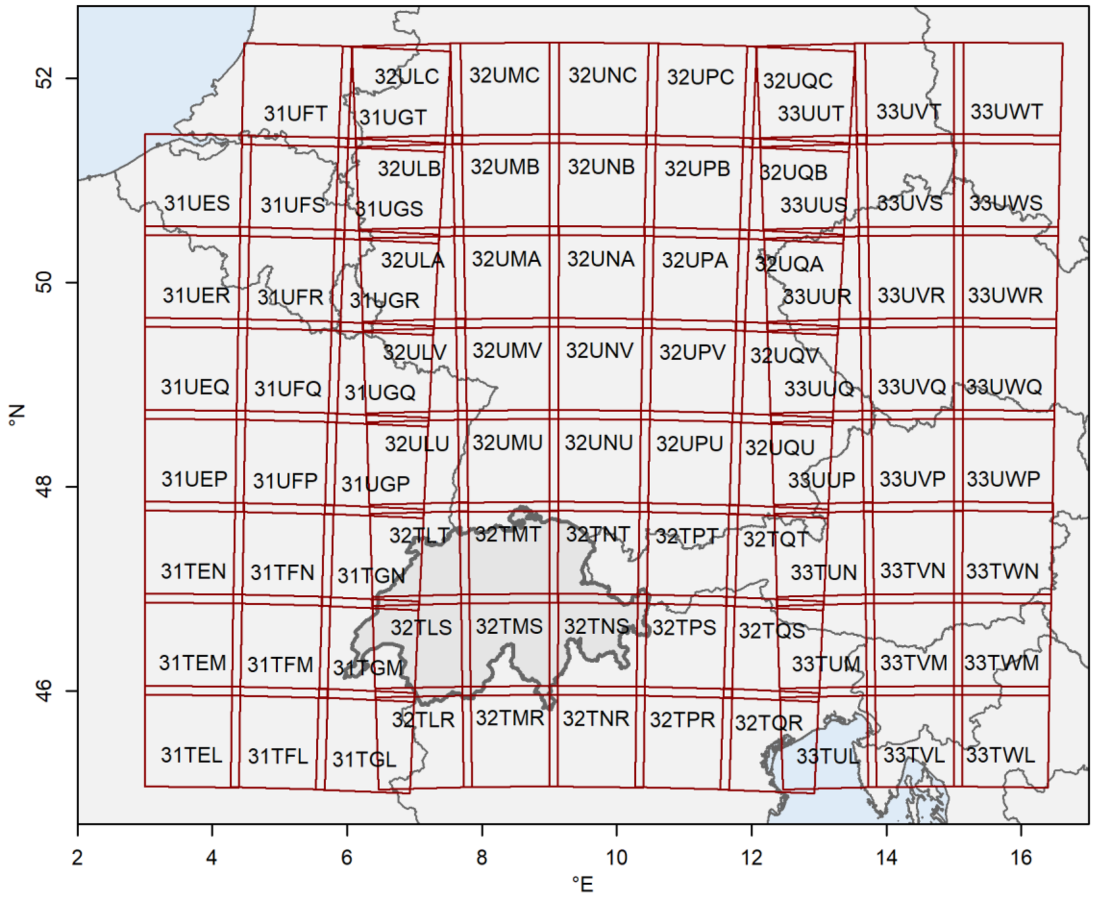

Fig. S3 | Sentinel-2 tiles analyzed. Patterns of early wilting were analyzed across all Sentinel-2 tiles depicted. The focal area of Switzerland is delineated with bold lines.

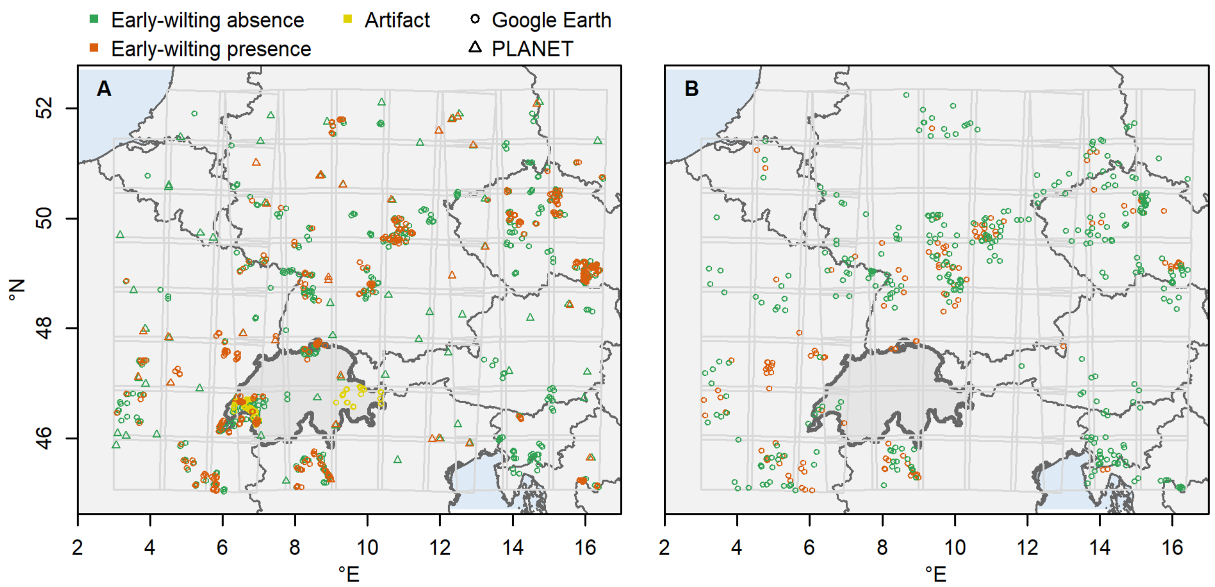

Fig. S4 | Distribution of visually interpreted training and test data. Distribution of training data polygons is shown in panel *A*; distribution of test data points is shown in panel *B*. Colors indicate early-wilting absence (green), early-wilting presence (brown), and artifacts, i.e., wilted grass, bare soil, logging activities (yellow). Locations interpreted with Google Earth imagery are shown as circles; locations interpreted with PLANET imagery are shown as triangles. Grey squares represent the Sentinel-2 tiles analyzed.

Table S1 | Summary of training and test data sets resulting from visual interpretation.

	Region	Class	Polygons (#)	Area (km^2)	Pixels (#)
Training data	Google Earth imagery based	Early-wilting presence	401	1.328	13’276
		Early-wilting absence	407	17.613	176’129
		Artifact (wilted grass, bare soil, logging activities)	74	0.356	3560
	Spatially stratified	Early-wilting presence	54	0.942	9418
		Early-wilting absence	86	10.019	101’904
Test data	Google Earth imagery based	Early-wilting presence	-	0.021	209
		Early-wilting absence	-	0.050	501

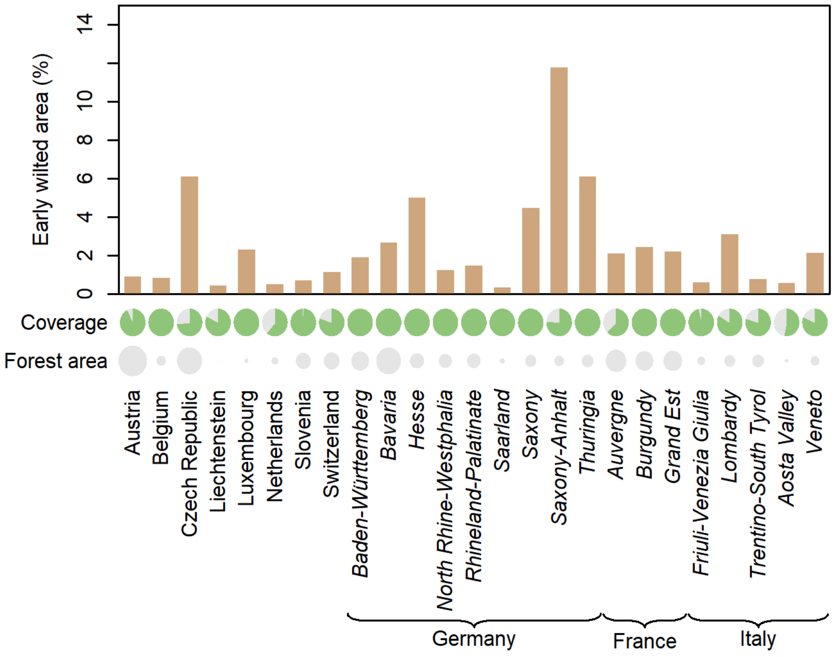

Fig. S5 | Fractions of pixels with predicted early-wilting presence for the main political units in the area. ‘coverage’ indicates the fraction of forest pixels that was covered by the analysis (only regions with >50% coverage are shown) and ‘forest area’ indicates the relative forested area in each unit.

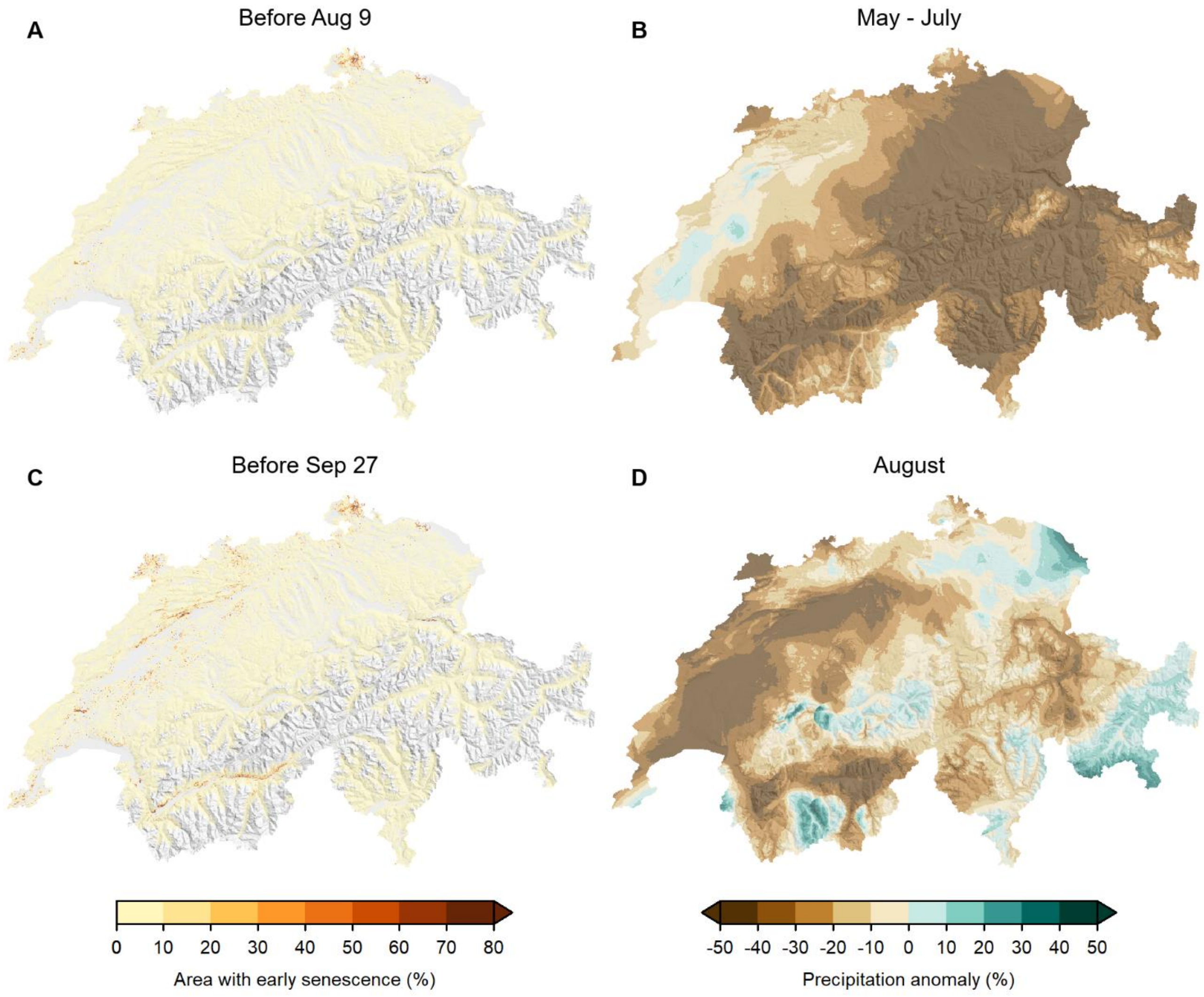

Fig. S6 | Fractions of early-wilted forest and preceding precipitation anomalies in Switzerland. Fractions of early-wilted forest with change points of the "Change, Aftereffect, Trend" analysis (see Methods) before August 9 are shown in panel *A*; fractions of early-wilted forest with change points before September 27 are shown in panel *C*. Panels *B* and *D* show 2018 precipitation anomalies relative to the period 1981-2010 for the May-July and August, respectively.

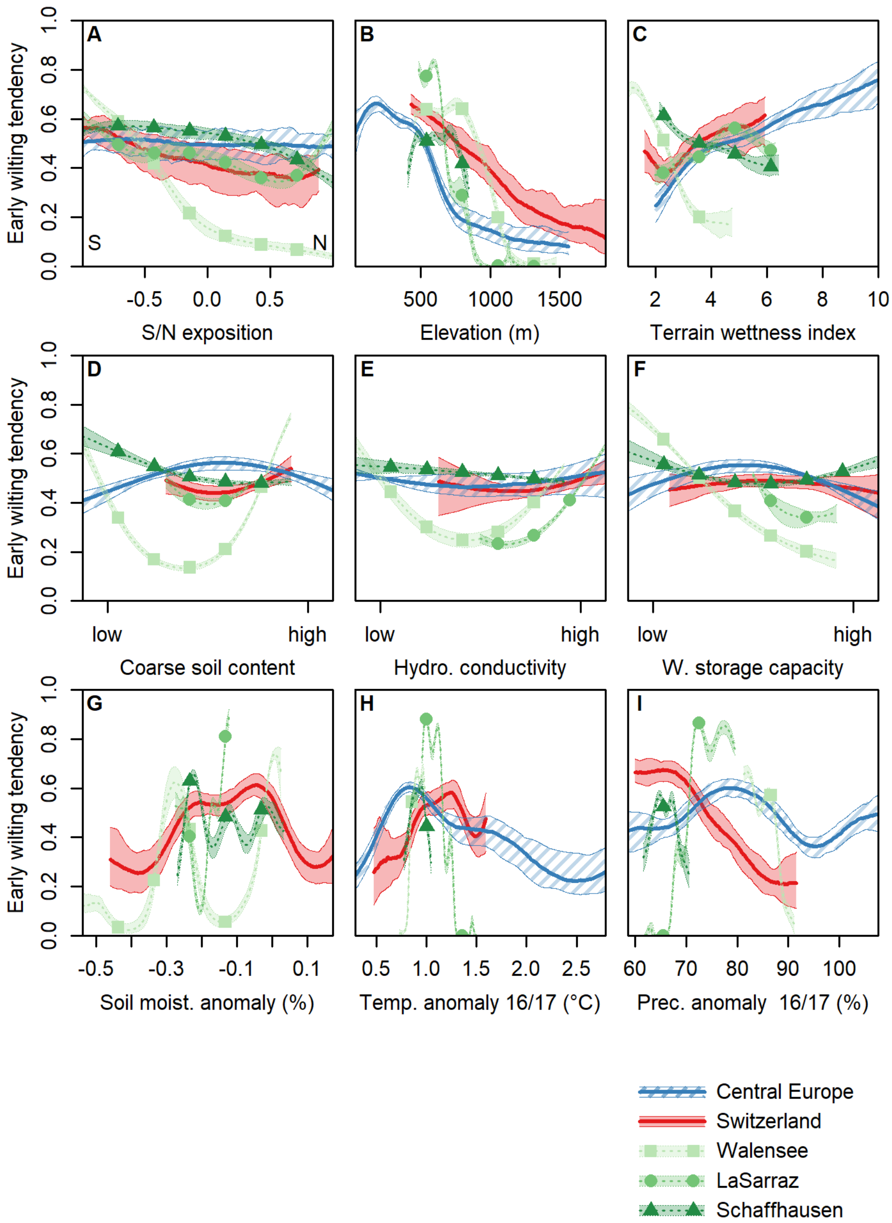

Fig. S7 | Univariate response curves from different regions/at different scales for additional predictors considered. Central lines represent median estimates among 100 models fitted on resampled data; polygons represent 95%-confidence intervals. For distinction of regions/scales see legend at the bottom.

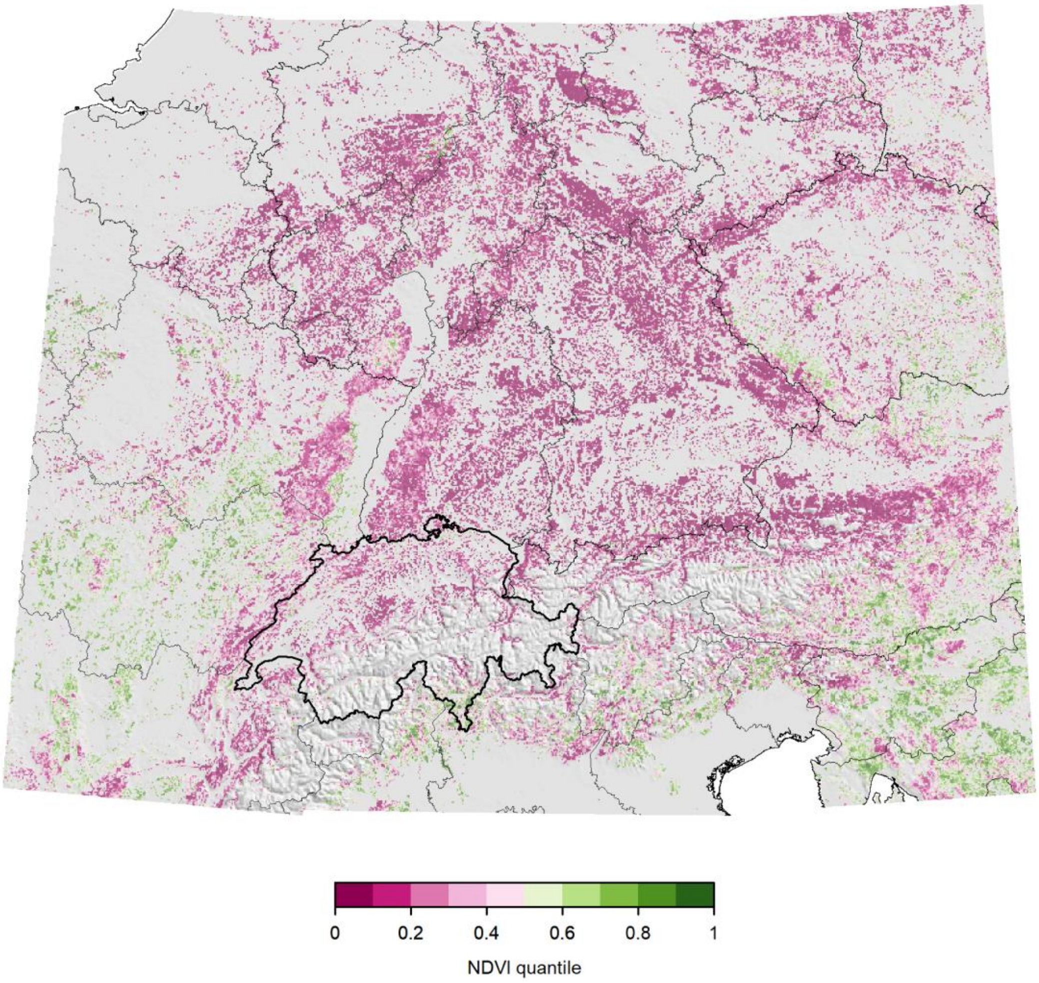

Fig. S8 | NDVI quantiles seen by MODIS. NDVI quantiles between mid-July and mid-September 2018 relative to the period 2000-2018. The quantiles have been derived from the 1000×1000m "MOD13A3" product from the MODerate resolution Imaging Spectrometer (MODIS) program. Illustrated are pixels with at least 30% forest coverage.

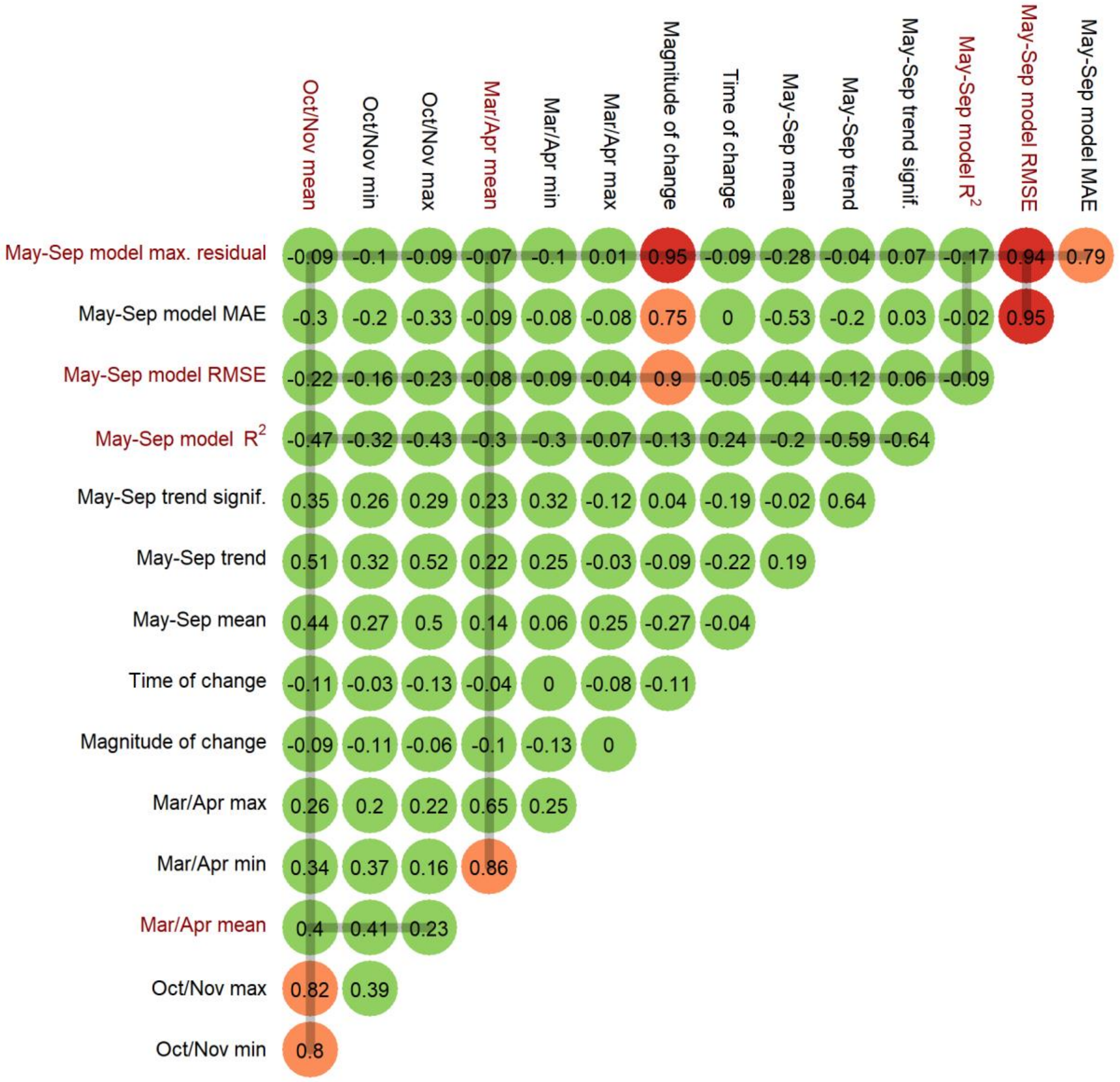

Fig. S9 | Pearson correlation coefficients between potential predictors of early-wilting presence in the training data. Green circles represent absolute Pearson correlation coefficients <0.7; orange circles represent absolute Pearson correlation coefficients between 0.7 and 0.9; and red circles represent absolute Pearson correlation coefficients higher than 0.9. Variable names in dark red and corresponding black-transparent lines represent variables that were removed in order to reduce multicollinearity. RMSE represents root mean squared error; and MAE stands for mean absolute error.

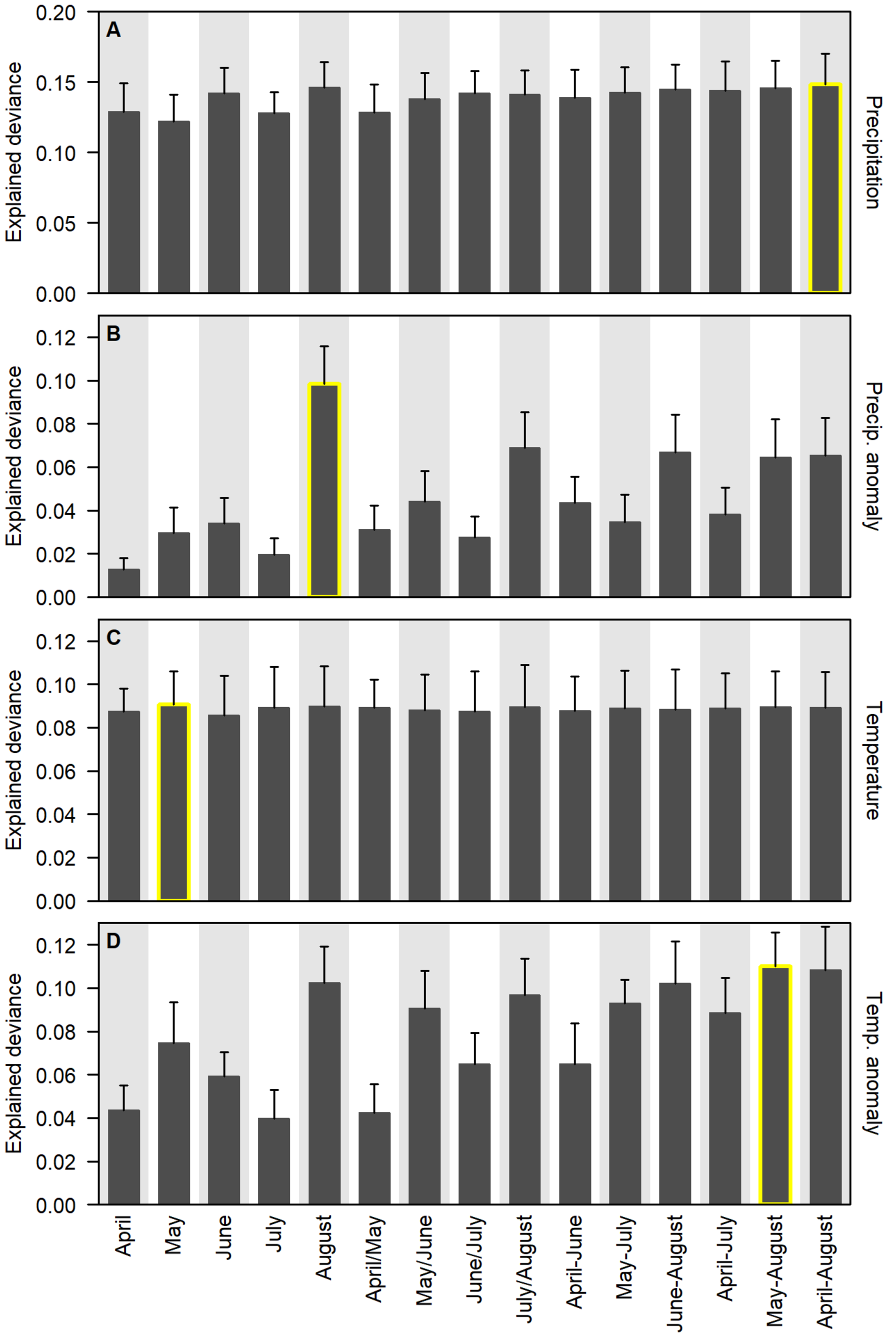

Fig. S10 | Explained deviance of climate predictors for different periods at the Central European scale. Univariate explained deviance of early-wilted forest are shown for precipitation (*A*), 2018 precipitation anomalies (*B*), maximum temperature (*C*), and 2018 temperature anomalies (*D*) for different periods between April and August. Bars indicate median values of 100 models fitted on resampled data; error bars indicate the 95%-confidence intervals. Bar of highest explained deviance for each predictor is highlighted with a yellow border.

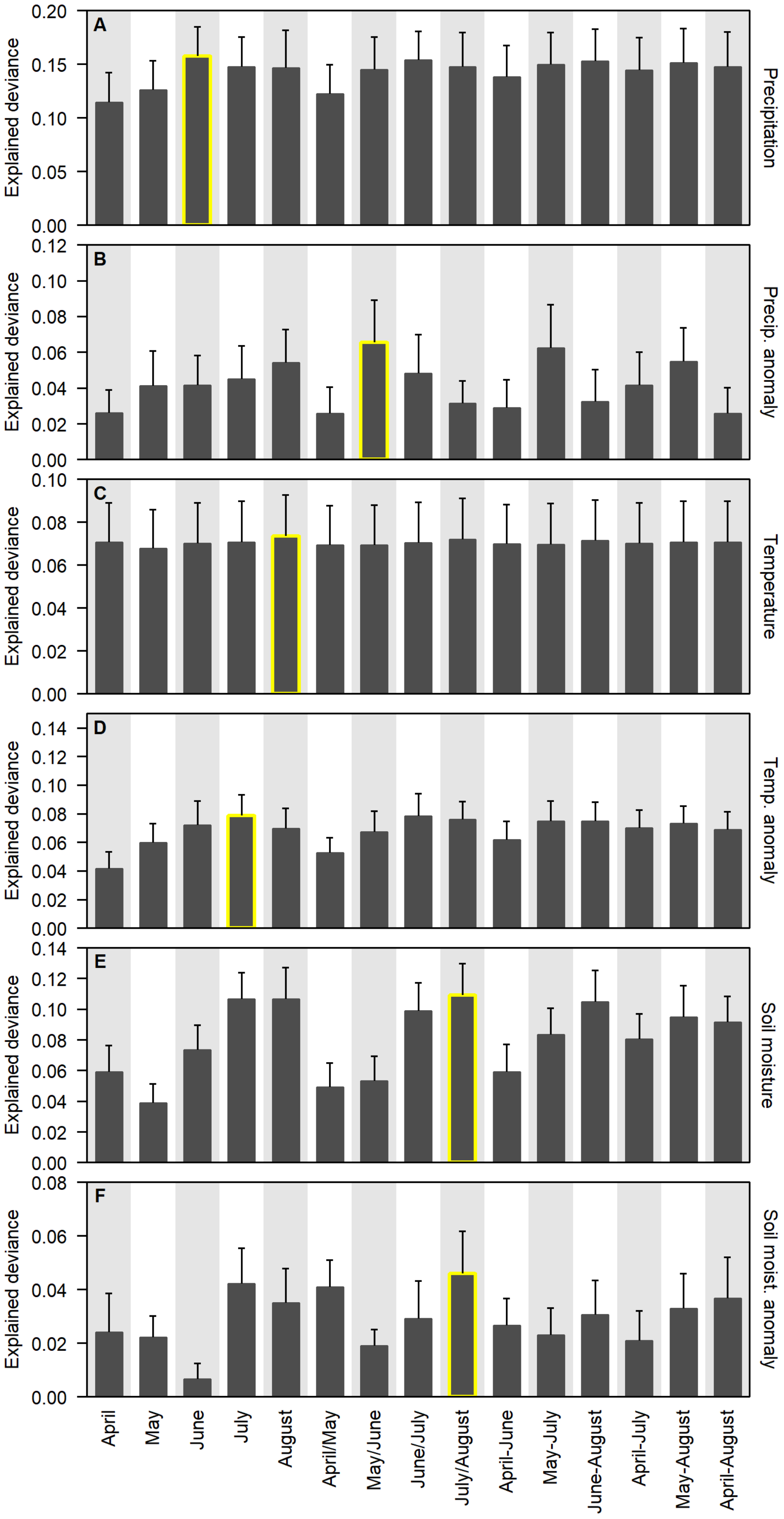

Fig. S11 | Explained deviance of climate and soil moisture predictors for different periods at the Swiss scale. Univariate explained deviance of early-wilted trees are shown for precipitation (*A*), 2018 precipitation anomalies (*B*), maximum temperature (*C*), 2018 temperature anomalies (*D*), soil moisture (*E*), and 2018 soil moisture anomalies (*F*) for different periods between April and August. Bars indicate median values of 100 models fitted on resampled data; error bars indicate the 95%-confidence intervals. Bar of highest explained deviance for each predictor is highlighted with a yellow border.

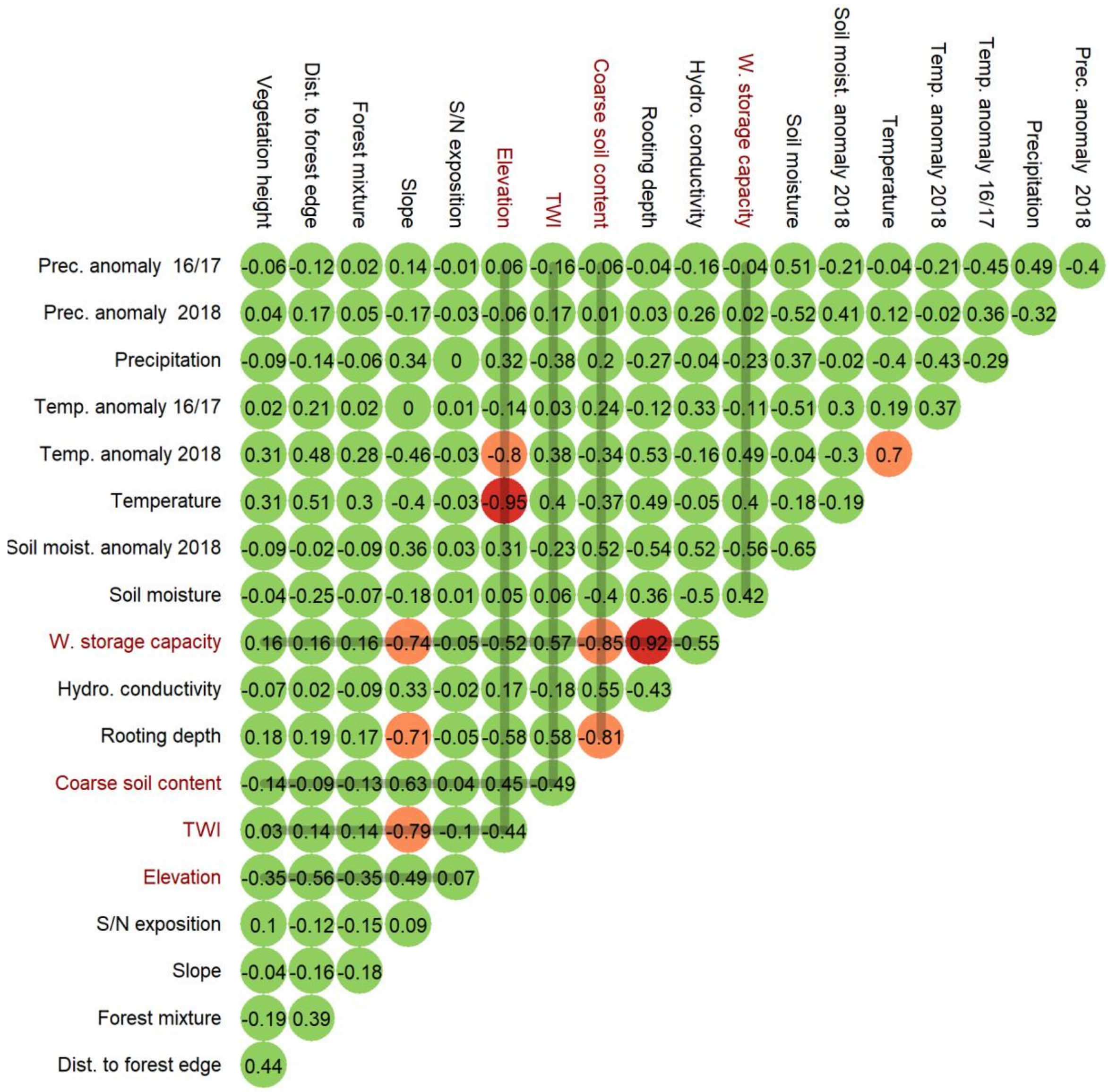

Fig. S12 | Pearson correlation coefficients between potential predictors used for the environmental GAM models and indication of removed predictors due to high correlation. Green circles represent absolute Pearson correlation coefficients below 0.7; orange circles represent absolute Pearson correlation coefficients between 0.7 and 0.9; and red circles represent absolute Pearson correlation coefficients higher than 0.9. Variable names in dark red and corresponding black-transparent lines represent variables that were removed due to high collinearity. Data basis were predictors at the Swiss scale. TWI represents terrain wetness index.

Supplementary Methods

Media analysis

We screened a large part of the German-speaking newspaper and magazine articles published in Switzerland during 2018 for keywords related to (1) drought, (2) forests/trees, (3) water stress/wilting responses. Articles were ordered from the media observation platform Swissdox (https://pro.swissdox.ch/, order number RE-1900823 made on March 11, 2019). We preprocessed all articles by removing punctuation, transforming all letters to lowercase, and replacing umlauts with the corresponding base vowels followed by an 'e'. Then, we evaluated the following criteria to test whether the topics drought, forests/trees, and water stress/wilting responses were mentioned in the articles: for the drought topic we expected the word 'trockenheit' (dryness) or the word part 'duerr' (drought) or both in the article; for the trees/forests topic at least one of the word parts 'baum', 'baeume' (trees), 'wald', 'waelder' (forests), or 'buche' (beech) had to occur; and for the water stress/wilting topic we expected at least one of the word parts 'verfaerb' (discoloring), 'verbraeun' (browning), 'mortal' (mortal), 'sterben', 'stirb', 'starb', 'storb', 'toedl', 'toet' (dying/dead), 'blattfall' , 'blattverlust', 'laubfall', 'entlaub' (leaf shedding), 'verdurst' (dying of thirst), 'vertrock', 'verdorr' (drying out), 'wassermangel' (lack of water), or 'welke' (wilting).

Supplementary Results

Selection of NDVI time-series statistics

The different analyses of NDVI time series resulted in 39 summary statistics in total of which we selected ten. We removed all statistics describing number of observations (five) and intercepts of time-series trends (three) as they had no direct link to early wilting. Due to insufficient coverage, we further removed all statistics describing trends in the sub-series before and after the change points detected in the "Change, Aftereffect, Trend"-analysis of the May-September subset (14 statistics). Similarly, we removed standard deviations for the March/April and the October/November periods due to insufficient coverage. In order to limit multicollinearity, we removed another five predictors (Fig. S9). These primarily included statistics describing uncertainty in the May-September linear trend model (R^2, root mean squared error, maximum absolute residual), as well as the average NDVI levels in March/April, and October/November. In the final predictor set, one variable combination with a moderate absolute Pearson correlation coefficient of 0.75 was left: mean absolute error of the May-September linear trend model and magnitude of change at the change point. Since we considered both of these predictors relevant for predicting early wilting, we kept the combination.

Univariate explained deviance of climate predictors for different periods

The explained deviance of the climatic drivers for different time periods varied in particular for climate anomalies and was not always consistent between the Swiss and the Central European scales (Figs S11 and S12). This is likely because the drought varied in its temporal evolution across Central Europe. However, in order to keep the period consistent for the different predictors and to account for hypothesized ecological relevance, we decided for August 2018 as the most relevant period for all climate predictors, except climatological precipitation, which we considered cumulated for the April-August period.

Correlation between climate, soil, terrain, and vegetation predictors

Among the environmental predictors considered, we found elevated absolute Pearson correlation coefficients particularly between elevation and temperature, as well as between terrain wetness index (TWI), slope, and soil conditions (Fig. S12). In order to reduce multicollinearity among predictors in multivariate analyses, we therefore removed elevation, TWI, coarse soil content, and water storage capacity from the predictor set. We removed elevation as it is a proxy for precipitation and in particular temperature and does not affect trees directly. Terrain wetness index was removed as its effect is slightly less straightforward to interpret than the effect of slope. Water storage capacity was removed due to its similarity to rooting depth. Among these two factors rooting depth appeared to be the simpler and more robust measure. Finally, we removed coarse soil content because its effect on water stress appeared less direct than the effects of rooting depth and hydraulic conductivity. However, univariate analyses were unaffected by these removals and early-wilting responses to all predictors are shown in Figs. 4 and S7.

References

García-Herrera, R., Garrido-Perez, J. M., Barriopedro, D., Ordóñez, C., Vicente-Serrano, S. M., Nieto, R., … Yiou, P. (2019). The European 2016/17 Drought. *Journal of Climate*, *32*(11), 3169–3187. https://doi.org/10.1175/JCLI-D-18-0331.1
